# Combining in-situ simulation and live HEMS mission facilitator observation: a flexible learning concept

**DOI:** 10.1186/s12909-021-03015-w

**Published:** 2021-11-15

**Authors:** Per P. Bredmose, Jostein Hagemo, Doris Østergaard, Stephen Sollid

**Affiliations:** 1 Department of Research, Norwegian Air Ambulance Foundation, Oslo, Norway; 2 Division of Prehospital Services, Air Ambulance Department, Oslo University Hospital, Postboks 414, Sentrum, 0103 Oslo, Norway; 3 Faculty of Health Sciences, University of Stavanger, Stavanger, Norway; 4 Institute of Clinical Medicine, University of Oslo, Oslo, Norway; 5 Copenhagen Academy for Medical Education and Simulation, Capital Region of Denmark and Department of Clinical Medicine, University of Copenhagen, Copenhagen, Denmark

**Keywords:** Simulation, In-situ simulation, Debriefing after observed missions, Education, HEMS, Prehospital

## Abstract

**Background:**

Continuous medical education is essential in Helicopter Emergency Medical Services (HEMS). In-situ simulation training makes it possible to train in a familiar environment. The use of a dedicated facilitator is essential; however, when an in-situ simulation training session is interrupted by a live mission, the efforts invested in the training are left unfulfilled. This study aims to evaluate if HEMS mission observation and debriefing by the simulation facilitator is a feasible alternative to mission-interrupted simulation training, and how this alternative to simulation training is perceived by both facilitators and HEMS crew members.

**Methods:**

Facilitator observation during live missions and post-mission debriefing was offered as an alternative to mission-interrupted simulation training over a one-year period at three HEMS bases. Immediate feedback was requested from crews and facilitators after each observed live mission on a predefined questionnaire. At the end of the study period, semi-structured interviews were performed with a sample of HEMS crew members and facilitators to further explore the experience with the concept. Numerical data about the sessions were recorded continuously.

**Results:**

A total of 78 training sessions were attempted, with 46 (59%) of the simulations conducted as planned. Of the remaining, 23 (29%) were not started because the crew had other duties (fatigued crew or crew called for a mission where observation was inappropriate/impossible), and 9 (12%) training sessions were converted to observed live missions. In total, 43 (55%), 16 (21%) and 19 (24%) attempts to facilitate simulation training were undertaken on the three bases, respectively. The facilitators considered mission observation more challenging than simulation. The interviews identified local know-how, clinical skills, and excellent communication skills as important prerequisites for the facilitators to conduct live mission observation successfully. Participating crews and facilitators found simulation both valuable and needed. Being observed was initially perceived as unpleasant but later regarded as a helpful way of learning.

**Conclusion:**

Live mission observation and debriefing seems a feasible and well-received alternative to an in-situ simulation program in HEMS to maximise invested resources and maintain the learning outcome. Furthermore, additional training of simulation facilitators to handle the context of live mission observation may further improve the learning output.

**Supplementary Information:**

The online version contains supplementary material available at 10.1186/s12909-021-03015-w.

## Background

Continuous professional development in medicine is essential to maintain competence and quality of care. Simulation training has a central role in combining experienced-based learning with a safe and patient independent learning environment [1]. In-situ simulation-based training combines a familiar working environment with simulation technology, thus providing safe and possibly time-effective training that has been shown to reduce mortality [2]. We have previously shown that in-situ simulation-based training is feasible even in an environment with an unpredictable and heavy workload, such as helicopter emergency medical services (HEMS) [3]. A prerequisite for implementing this training concept is positive acceptance by the crews and a devoted and appropriately skilled facilitator [3, 4]. A major obstacle for implementation, however, is the relatively large number of in-situ simulations that are interrupted or never started due to actual HEMS missions [3]. These interruptions will mostly leave the invested time and resources in training untapped with the potential of demotivating the facilitators and consequently disrupt the successful implementation and maintenance of the in-situ simulation training program. Ways to overcome this barrier must therefore be sought.

Observation of actual professional conduct as a means for improved performance has long been used in aviation, where the observation of the cockpit performance during actual flights by a third-party instructor is an integrated part of the professional development of pilots [5]. We speculate that a similar concept, where the facilitator joins the mission interrupting the planned simulation as an observer and subsequently governs a post-mission debriefing, could be a training opportunity. In this way, the facilitator’s availability and competence could be used while maintaining a safe learning environment for the crews, similar to the in-situ simulation training. However, no previous studies have evaluated the use of a facilitator as a live mission observer to our knowledge.

This study aims to evaluate the feasibility of conducting live mission observation and post-mission debriefing by a simulation facilitator as an alternative to planned but interrupted in-situ simulations and how the facilitators and crew members perceive this alternative training format.

## Methods

To explore the possible benefit of introducing live mission debriefing as a proxy for the missed in-situ simulation and debriefing, we used a mixed-methods design [6–8]. Our triangulation included a prospective observational data collection to quantify and describe the number and types of intervention (i.e. simulation or live mission observation), a questionnaire to collect the facilitators’ and crews’ immediate experience of the training, and interviews for in-depth information about the experience. In our study, all data collection methods were given the same priority, although used partly in parallel and partly in a sequential manner.

### Norwegian HEMS system

The Norwegian HEMS is a national service funded by the government. At the time of the project, two private companies were contracted to provide helicopters, pilots and HEMS technical crew members (HTCM). The standard crew configuration consists of a certified anaesthesiologist, an HTCM and a pilot. One base also includes an anaesthetic nurse in the crew at the time of the study. All HEMS bases respond to primary medical and trauma missions, day and night, and perform interhospital transports and search and rescue (SAR) missions. The organisation of the Norwegian HEMS has been described in detail in a previous article [9].

### Study population

To achieve a representative sample of HEMS bases in Norway for our study, we recruited three bases with different mission profiles for the adapted simulation program. The base at Lørenskog covers a densely populated area and is the busiest HEMS base in Norway in terms of the number of missions. The base at Ål is located in the mountain region of South Norway with a low population density. The base at Ålesund is located in South West Norway’s coastal region with a mixed population density and is the only HEMS base in Norway with a 4- crew configuration. In the study period, the flight operation at Ålesund HEMS was run by a different company than at the other two bases. All bases operate 24 h a day, all week, all year around. The number of annual missions for the bases at Lørenskog (two helicopters), Ålesund and Ål are approximately 1900, 650 and 600, respectively [10].

### Study design

The study was conducted in two stages. The first stage was a prospective multicentre study with observational data collected between May 19th, 2016, and May 18th, 2017. In-situ simulation training was offered as described in a previous study from an earlier stage of the project [3, 9]. The facilitators developed scenarios tailored to each base depending on each base mission profile. The in-situ simulation could be located indoor or outdoor and would involve all members of the crew. The training at each HEMS base was run by one or two experienced senior HEMS physicians selected by the lead physician at the HEMS base to be facilitators [9]. The facilitators were trained according to the EUSim simulation facilitator course [9, 11]. The facilitators were only responsible for the training at their own base and received remuneration for simulation training outside their ordinary hours of work at the HEMS base. The training was planned by the local facilitator on a convenience basis and took place during the daytime on weekdays. There were no requirements or expectations regarding the total number of sessions or their frequency during the study period. The facilitators emphasised to the crews that the training was an optional learning and training opportunity, rather than a compulsory task. In cases where the planned simulation was interrupted by live missions the facilitator could opt to join and observe the mission if appropriate. Crews were informed verbally and in writing, that the in-situ simulation could be changed to an observed live mission with debriefing if the in-situ simulation was interrupted. The debriefing after both in-situ simulation and live mission observation was performed using the PEARLS framework which is structured as; reaction, description, analysis, application/summary [12].

In the second stage of the project, structured individual interviews were conducted with selected crew members from the three bases that participated and the facilitator at each base to explore their experiences with HEMS mission observation as an alternative to the in-situ simulation. The second stage took place between June 1st and June 15th, 2017.

### Data collection and analysis

#### Observational data

The facilitator collected data on the duration of the in-situ simulation and classified it as completed with debriefing or interrupted by a mission call-out with or without subsequent live mission observation. Data was instantly recorded on a premade paper form by the facilitator and later entered into an electronic database (Questback Essentials, Oslo, Norway). This paper form was designed by one author (PB) and approved by the other authors. It was designed for a practical purpose and collecting data about practicalities of the simulation including information about cancellations. The form was completed after the training and subsequently entered into the database. No data related to individual crew members was recorded. We summarised continuous data using median (quartiles) and categorical data as numbers (percentages) and compared non-paired observations with the Mann-Whitney U test. All statistics were calculated using SPSS (IBM Corp. SPSS Statistics for Windows, Version 25, IBM Corporation, Armonk, NY, USA).

#### Questionnaires

Following both in-situ simulation training and observed live mission debriefing, the participating crew and the facilitator rated their degree of satisfaction with the session on a visual analogue scale (VAS) from 0 to 100 mm, where 0 represented completely unsatisfactory and 100 represented maximum satisfaction [13]. The participating crew members were then asked to respond to one of two questionnaires with 14 questions, adapted to either training as planned with an in-situ simulation or observed live mission with a debriefing. All answers had to be indicated on a 7-point Likert scale where 1 corresponded to “*I strongly agree* “and 7 to “*I stro*ngly disagree” [14]. The questionnaires, to capture the participants evaluation of the simulation-based training, were similar to the questionnaire used at a previous stage of this project but adapted to the current context [3]. An initial draft was made by two of the authors (PB and SS). The questionnaire was then reviewed by a pilot, a HTCM and a physician. The modified questionnaires were piloted and reviewed by two separate full HEMS crews from the Lørenskog HEMS base to ensure that the questions were clear and comprehensible. An English version of the questionnaires, translated independently by two authors (PB and SS) is available as Additional file 1.

#### Interviews

Following the one-year study period, we invited a group of physicians, HTCMs and facilitators to take part in a structured individual interview. Interview candidates were chosen so that all participating bases were represented. We did not include the pilots in these interviews since they only had a minor role in the patient-centred part of the missions. An interview guide was used (Additional file 2), but interviewees were encouraged to elaborate and discuss other topics that would emerge during the interview if they wanted to. All interviews were done on a weekday during daytime and lasted approximately 20 min each. The interviews were undertaken via telephone for practical reasons and by the first author (PB) exclusively. The interviewer is experienced both as a consultant in prehospital critical care and as a simulation facilitator. He has conducted interviews previously and has conducted studies on in-situ simulation in HEMS services demonstrating a positive effect on training culture [3, 9]. When no further information emerged on a topic, the interview would continue according to the interview guide. At the end of the interview, the interviewees were encouraged to mention anything they felt had not been addressed. The interviews were recorded on two separate digital recorders for proper redundancy in case of technical difficulties or quality issues with the recordings. The digital interview files were stored on an encrypted server to which only the first author (PB) was granted access.

The recorded interviews were transcribed verbatim by a medical student who received an hourly payment for the job and was not part of the project. The primary author (PB) compared the transcriptions to the recorded interviews to ensure the transcription’s quality and accuracy. All digital recordings of the interviews were deleted after analysis.

The transcribed interviews were analysed according to Malterud’s “Systematic text condensation” by two of the authors (DØ and PB) [15, 16].

To obtain a total impression of the interviews, the main author (PB) read through the transcripts to get a sense of the interviews and identify themes. The themes were applied if appropriate by two authors. The interviews were then reread to define and identify “meaning units” covering the themes identified in the previous step. These meaning units were text fragments/quotations with information about the participant’s thoughts. The meaning units were then sorted into subthemes and then synthesised into text. After synthesising, the interviews were reread through to ensure that no information was lost.

## Results

### Descriptive observational data

A total of 78 training sessions were attempted, with 43 (55%), 19 (24%) and 16 (20%) attempts at each of the three bases, respectively. In-situ simulations were completed as planned in 46 (59%) of the cases. In 23 (29%) cases, the simulations were not started because the crew had other duties such as daily aircraft and equipment checks or administrative tasks to attend. In nine (12%) of the simulation attempts, a live mission interrupted the training. All these situations were converted to live mission observations with debriefing (Table 1).

**Table 1:** The number of attempted in-situ simulations and live mission observations summarised for each participating base

Name of the base	In-situ simulation conducted with no interruption	In-situ simulation attempted but not conducted	In-situ simulations changed to live mission observations
Lørenskog	8	4	4
Ål	14	5	0
Ålesund	24	14	5
Total	46	23	9

### Questionnaires

The overall satisfaction with the in-situ simulations and the observed live missions was high, with no differences in VAS scores between the two (Table 2). All crewmembers found both in-situ simulation and observed live missions had an appropriate duration, and that relevant standard operating procedures (SOPs) were included in the in-situ simulation and the debriefings after missions. The answers were within the two most positive scores except for two questions; Following the in-situ simulations, most participants indicated that they felt uncomfortable exposing their skills and competencies while being observed during the in-situ simulation training and that the in-situ simulation training was interfering with “non-mission related” duties. Interestingly, the corresponding questions following the live mission observations scored the completely opposite. For observed missions, the participating crews neither expressed concerns about exposing skills and competencies nor did they indicate that it interfered with non-mission related tasks. Data from the questionnaires are presented as medians with quartiles in Table 3.

**Table 2:** Visual analogue scale (VAS) scores expressing to what degree the crewmembers were satisfied with the in-situ simulation and live mission observations summarized with means and standard deviation (SD) for each crew member group, with 0 mm representing the least of satisfaction and 100 mm representing the most of satisfaction

Crew member	In-situ simulation and debriefing		Live mission observation and debriefing		*P*
	*n*	Mean VAS score (mm) ± SD	*n*	Mean VAS score (mm) ± SD	
Physician	46	86 ± 10	9	88 ± 14	0.765
HTCM	46	91 ± 7	9	93 ± 4	0.952
Pilot	45	87 ± 11	7	87 ± 11	1.0
Nurse	24	92 ± 4	5	91 ± 10	0.339

*HEMS* Helicopter Emergency Medical Service, *HTCM* HEMS technical crew member

**Table 3:** The participant’s evaluation of the training on a 7-point Likert scale, where 1 represent “I strongly agree”, and 7 represent “I strongly disagree” (Median scores with quartiles) (*Obs* observation, *Sim* simulation)

Modality	Question	Physician		HTCM		Pilot		Nurse	
		Median (quartiles)	n	Median (quartiles)	n	Median (quartiles)	n	Median (quartiles)	n
Obs	Sufficient time was allotted for debriefing and feedback after the mission observation	1(1–1.5)	9	1.5(1–2.75)	8	1(1–2)	7	1(1–1)	5
Sim	Sufficient time was allotted for crew simulation training	1(1–2)	46	1(1–2)	46	1(1–1)	44	1(1–1)	4
Obs	The live mission observation was completed without interrupting other on-call duties	1(1–2)	9	1.5(1–2)	8	2(1–2)	7	1(1–3)	5
Sim	The simulation training was completed without interrupting other on-call duties	7(5–7)	46	6(3.75–7)	46	7(1–7)	45	7(7–7)	4
Obs	I am comfortable with being observed by a peer during a live mission	1(1–2)	9	1(1–1)	8	1(1–1)	7	1(1–1)	5
Sim	There was enough equipment available to make the simulation training realistic	2(1–2)	46	2(1–2)	46	1(1–2)	45	1(1–2)	4
Obs	I felt comfortable with the way the live mission observation was carried out	1(1–2)	9	1(1–1)	8	1(1–1.25)	6	1(1–1.5)	5
Sim	I felt comfortable with the way the simulation training was set up	1(1–1)	45	1(1–1)	45	1(1–1)	44	1(1–1)	4
Obs	I felt comfortable with exposing my skills and competencies during a live mission	2(1–3.5)	9	1(1–1)	8	1(1–1)	7	1(1–1)	5
Sim	I felt comfortable with exposing my skills and competencies during the simulation training	7(2.75–7)	46	6(2–7)	45	7(1–7)	45	7(7–7)	4
Obs	Live mission observation with debriefing and feedback gives me the same benefits as simulation training	1(1–4.5)	9	2(1–4.25)	8	2(1–2)	7	1(1–3)	5
Sim	Simulation was a realistic way to train	1(1–2)	46	1(1–2)	45	1(1–2)	45	1(1–1)	4
Obs	The mission characteristics were well suited for mission observation with consequent debriefing and feedback	2(1–3)	9	1.5(1–2	8	1(1–2)	7	1(1–2)	5
Sim	The topic of the simulation training is relevant for this kind of simulation training	1(1–1)	45	1(1–1)	46	1(1–1)	44	1(1–1)	4
Obs	Live mission observation with feedback and debriefing is useful for my occupational category	1(1–1)	9	1(1–1.75)	8	1(1–2)	7	1(1–1)	5
Sim	This type of simulation training is useful for my occupational category	1(1–2)	44	1(1–1)	45	1(1–2)	44	1(1–1)	4
Obs	The facilitator managed to create a learning environment by relating elements from the mission observation to our SOPs	1(1–1.5)	9	1(1–1)	8	1(1–2)	7	1(1–2.5)	5
Sim	The simulation scenario was representative of the SOPs we trained on	1(1–2)	46	1(1–2)	46	1(1–2)	45	1(1–1.75)	4
Obs	The execution of the mission was not disrupted by the peer joining for mission observation	1(1–2)	9	1(1–1)	7	1(1–2)	7	1(1–1)	5
Sim	The topic of the scenario training was relevant to the mission profile of the base	1(1–1.5)	45	1(1–1.5)	45	1(1–2)	44	1(1–1)	4
Obs	The debriefing and feedback after the mission observed was useful	1(1–1)	9	1(1–1)	9	1(1–2)	7	1(1–1)	5
Sim	The feedback after the simulation training was useful	1(1–1.25)	46	1(1–2)	45	1(1–2)	45	1(1–1)	4
Obs	Enough time was allotted for debriefing and feedback after the mission observation	1(1–1.5)	9	1(1–2)	9	1(1–2)	7	1(1–1)	5
Sim	Enough time was allotted for feedback after the simulation training	1(1–1.5)	45	1(1–2)	45	1(1–1)	45	1(1–1)	4
Obs	It was easy to motivate oneself to complete the mission with mission observation	1(1–1.5)	9	1(1–1)	9	1(1–2)	7	1(1–1)	5
Sim	It was easy to motivate oneself to complete the simulation training	1(1–2)	46	1(1–2)	45	1(1–1)	45	1(1–1)	4
Obs	I have a positive attitude towards this kind of training	1(1–1.5)	9	1(1–1)	9	1(1–1.25)	6	1(1–1)	5
Sim	I have a positive attitude towards this kind of training	1(1–1)	46	1(1–1)	44	1(1–1)	45	1(1–1)	4

### Interviews

The demographics of the participants in the interviews are shown in Table 4. Only two facilitators were able to participate in the interviews. The gender ratio has an overweight of male participants, which reflects the gender ratio in Norwegian HEMS services. As Table 4 shows the majority of the participants are experienced as HEMS crew members.

**Table 4:** Demographics of interviewees

Participant	Age (years)	HEMS experience (years)	Gender	Position in the project
1	43	7	Male	Physician
2	38	1	Male	Physician
3	39	2	Female	Physician
4	32	5	Male	HTCM
5	31	3	Male	HTCM
6	38	9	Male	HTCM
7	44	15	Male	Facilitator
8	31	7	Male	Facilitator

*HEMS* Helicopter Emergency Medical Service, *HTCM* HEMS technical crew member

In the interviews, we identified four recurring themes; the facilitator role, the training itself, the outcome of the training and comparison of in-situ simulation and observed missions.

The interviewed facilitators expressed that they considered live mission observation to be more challenging as a facilitator and that being in the proper role as an observer only was essential. The facilitators describe that the lack of preparation for the debriefing was a challenge. They also mentioned that a benefit of observing missions was the opportunity to debrief both rare events and routine missions. Prerequisites highlighted by the facilitators for a successful live mission observation and debriefing were: a background with local knowledge, clinical skills, and excellent communication skills. They further expressed scepticism towards facilitators evolving from the group of physicians who are also a part of the management group and that the availability of the facilitators could be a challenge.

Both facilitators and participating crews indicated that a frequency of training sessions ranging from weekly to monthly training sessions was ideal. All interviewed groups expressed a high degree of motivation for both in-situ simulation and observed live missions but also emphasize that the training concept requires support from the management.

Participating HTCM and physicians describe learning outcomes within the areas of both clinical skills and communication skills. Furthermore, they describe that debriefing leads to a perception of an overall improvement of the mission, ranging from the planning phase all the way to the end of the mission.

Although some interviewees initially perceived it as stressful to be observed, they describe it as being beneficial. Some also commented that being observed had an impact on the team dynamics. The interviewees express a need for both planned in-situ simulations and observed missions and that they both can play a role in training. In-depth analysis and results from the interviews are shown in Table 5, where both facilitators and crew members’ opinions and experiences are expressed.

**Table 5:** Results from the interviews of the crew members with code groups, subgroups, meaning units and quotations shown

Themes	Subthemes	Meaning units	Text condensation
**Facilitator**	Availability	Availability	Interviewees described the challenges to incorporate time to facilitate into a busy working schedule. 10 am is mentioned as a good time for starting training. One facilitator occasionally found it difficult to go onto a mission in the case that it would interfere with parents following their child during an incubator transport.
		Timing	
	Pedagogical	Pedagogical challenges	Participants describe joining a live mission as more challenging than conducting the simulation and feel that experience is vital for that role. It is also mentioned that it can be a challenge to comment on habits that colleagues have used for decades. On some occasions, facilitators felt it was difficult for ambulances to know whom to interact with.
		Experience	
	Role	Role	It is essential for the facilitator to be able to observe and not participate in the mission, and facilitators need to “know their place” and preferably wear an observer west. Important to keep the role as facilitator and not start any talk about the mission or simulation outside the participating crew.
		Trust	
	Background	Facilitator background	The facilitator does preferably have local knowledge, and it is even mentioned that several local HEMS physicians could be trained as facilitators and go on missions with others. For some, the thought of management or external facilitators riding along on a mission is frightening, whereas others suggest that this is a chance for external feedback. Overall, there is scepticism to having facilitators coming from the management group.
		Selection	
	Skills	Communication	Interviewees agree on the importance of right skills for the facilitator. This includes having a genuine interest, clinical experience and good communication skills as well as and local knowledge.
		Interest	
		Local knowledge	
**Training**	Frequency	Frequency of training	Some participants mention a training frequency of once a week, and at least once a month.
	Motivation	Motivation	Both facilitators and crew participants report a high level of motivation among the participating crews, although it did vary.
		Variation	
	Organisation	Organised into service	The support from the management is mentioned as necessary. There are wishes for getting such training organised into the Norwegian HEMS services. The general acceptance and priority of training are noted.
		Management support	
**Outcome**	Peer feedback	Peer feedback	Participants report the usefulness of feedback from a (participating/facilitating) colleague. This is seen as an exchange of experiences rather than top-down teaching that often has been practised in traditional teaching.
		Difficult cultures	
		Communication	
	Correction of habits	Impractical habits feedback	Some participants mention this as an arena where not only communication in general, i improved, but even tricky topics in a challenging crew composition are easier during simulation than during regular workdays. Since most HEMS crews are alone on a mission, this is seen as an opportunity to get feedback and suggestion for improvement of minor daily mission details.
		Everyday topics	
		Practicalities	
	Longitudinal learning	Longitudinal debrief	In this form for training, participants mention that it is a possibility that they do not receive feedback on all aspects of a mission. This includes not only the clinical part but also the planning phase and choice of equipment brought on the mission.
**Comparison**	Simulation	Artificial setting	It is described that it is easier to have a planned debriefing structure during the simulation training. More challenging to facilitate mission observation. The simulation gives rise to train rare events as well as optimising the handling of frequent challenges. The predictability of simulation regarding the debriefing topics is essential. The trust among the crew in simulation is mentioned. Simulation can feel more as an artificial setting.
		Simulate rare/frequent events	
		Debrief structure	
		Predictability	
	Mission	Anxiety	Some participants have been nervous before having someone observing them on a mission; however, they describe this as a positive experience regardless. This form of supervision is beneficial. It is found stressing but at the same time; they learned a lot. Some are more worried about exposure but like to get feedback. Participants meant this leads to improved performance of them as individuals and for the team.
		Performance improvement	
		Supervision	
		Planned vs unplanned	
		Peer feedback	
	Comparison	Choice	Interviewees agree on the usefulness of both simulation training as well as having one observing them on a mission, although it can feel intimidating initially. There are pros and cons to both training forms.
		anxiety	
		No difference	
		Different outcomes	
	Priority/Choice	Mission	Participants explain that they think both forms for of training have a role in the Norwegian HEMS services. Interviewees would like to have both forms of training integrated into the service. If to choose, some distinctively prefer having one accompanying them on a mission.
		Both forms	

## Discussion

In this study, we found that observed live missions can successfully be used as an alternative to in-situ simulations when live mission interrupts the planned simulation training. The observed mission concept was well received by the crews, and especially the debriefing from live missions was experienced as useful. The facilitator role was, however, more demanding during live missions due to the unpredictable nature of the missions and the consequent inability to plan both content and subsequent debriefing. The facilitator’s local knowledge, clinical skills and communication skills seem essential to create a good learning experience for the participating crews for both in-situ simulations and observed live missions.

Our primary motivation for evaluating the concept of live mission debriefing as an alternative to in-situ simulation was the realisation in previous studies that a relatively high number of in-situ simulation training sessions were interrupted by live mission callouts and eventually aborted [3]. By including the facilitator in the live missions, we hoped to maximise the use of the dedicated facilitators time spent preparing and executing the training and increasing the learning outcome for the crews. The facilitators did experience the observation and debriefing of live missions as more challenging than the in-situ simulations. In the interviews, the facilitators indicate that they felt poorly prepared for the live mission debriefing setting. This feeling seems to come from a mixture of unfamiliarity with the role as facilitator in this context, e.g. finding ones’ role in the mission, and the unpredictability of the mission and consequently the topics to be debriefed. The facilitators had all attended an instructor course for simulation facilitators; a course focused on facilitating learning from simulations and not real-life observation [11]. The debriefing techniques taught in the course might be more challenging to apply in the debriefing of a real-life observation, or the context they are taught in does not promote their generic use. We also speculate whether the challenge of debriefing the live missions is experienced as more challenging by the facilitators because they are less prepared to debrief the medical aspects. This in contrast to the in-situ simulation that has been prepared by the facilitator and allows the facilitator to be more immersed in the medical theory of the scenario. For instance, in the in-situ simulation, the facilitator knows the “condition” of the patient, whereas in live mission observation the facilitator only knows “what the crew see”, and the rest is assumptions. This means that the facilitator is unable to prepare for the session and may feel les prepared to provide high quality feedback. Still, the non-technical components in the debriefing will be the same as in the in-situ simulation. It, therefore, seems reasonable that facilitators need additional training to manage the specific contextual challenges of debriefing experienced colleagues after live missions and prepare the facilitator for the challenges of riding along on a live mission as an observer. This is something we did not consider in the preparation of the facilitators for the project.

The crew members interviewed further emphasised that the facilitator must be a skilled communicator and a HEMS physician, preferably from the local base. Having a clinically proficient facilitator able to share expertise with good judgment can be motivational [17]. The continuous development of facilitators for both simulation and observed live missions seems essential for the success of a concept like the one presented in this study, which others have stated [18, 19].

The contextual role of the facilitator is quite different in a live mission than in a simulation. The facilitator’s presence on the mission also raises some fundamental questions: should the facilitator be just a passive observer or available as a potential resource in the mission, and how does the facilitator presence influence the crews’ team dynamics? Is it e.g. ethically acceptable that the facilitator does not contribute to the mission if it would impact the patient’s outcome positively, and how would such a contribution influence the debriefing and the mission? The feedback from the interviewees is that the role of the facilitator in the mission must be clearly stated before the facilitator joins the mission.

In the interviews, it is mentioned by the facilitator that they experience there is a time of the day, which is the most suitable for in-situ simulation with the least risk of interruption. Adaptive behaviour is important for the sustainability of a project, as fewer interruptions are favourable.

The data from the questionnaire showed that crews found the two learning opportunities similarly helpful in terms of learning experience and overall satisfaction. Interestingly though, the in-situ simulations generated more discomfort regarding being observed by a peer than the live mission observations. One explanation could be that the crews found the in-situ simulation cases more challenging than the live missions. Intuitively one might think that being observed in real clinical practice would be more stressful than being observed in a simulation under controlled circumstances and with no risk of harming a patient. Participants’ perception of stress during simulation and observation is not unheard of. Taylor et al. found that paramedic students participating in learning-orientated simulation almost all expressed feelings like stress and anxiety after the simulation [20]. However, some adaptation occurs; in a study by Rosenzweig, emergency medicine residents were video recorded during patients encounters and reported a discomfort that diminished over time [21].

The crew also seemed to find the observed live mission concept less intrusive than simulations in that it did not interfere so much with other on-call duties. This makes sense since missions are part of the duties when on-call and making a learning experience out of it is an effective use of time. A similar finding where in-situ simulation was found to be interruptive has been published [22].

The scores were almost identically positive for the two ways of training. We speculate whether this is a sign of the crews’ need and desire for feedback on their performance. On most missions, the crews work without peer support and the possibility to receive peer feedback. With the facilitator joining the mission, feedback can be provided. Another explanation for the almost identically positive scores could be that the facilitators were skilled to perform both tasks satisfactorily, despite their expressions of challenges with live mission debriefing.

We have previously addressed the challenges of implementing in-situ simulation for on-call crews (unpublished data; “Challenges to the implementation of in situ simulation at HEMS bases: a qualitative study of facilitators’ expectations and solutions”, Bredmose et al.). The findings in this study confirm our previous finding that implementation is tightly coupled with the facilitator’s contribution and commitment, and support from the management, and it seems this also applies to live mission observations. The management must support both the in-situ simulation training and provide an organisational structure for the facilitators to conduct training [23].

## Discussion of the methods used

This study was limited to three HEMS bases in Norway. By involving more bases in the study, we may have achieved a higher number of observed live missions that may have influenced our experience with the concept. On the other hand, the three bases represented the typical profiles of Norwegian HEMS, and one of the bases is the busiest HEMS base in Norway in terms of the numbers of missions. Given the explorative design of this study and the mixed-method approach, we think the study identify valuable challenges with the concept of in-situ simulations and observed live mission that will be useful in the future development of the concept. Although we experienced saturation in the answers in the interviews, the number of participants was limited. A larger group of interviewees might have given a broader insight into the topic, as would more observed live missions.

The use of facilitators as debriefers after live missions is, in our view, an interesting concept that requires more investigation. Our results provide an initial experience and identify areas of improvement that should be the basis for further studies. The concept may even prove to be helpful in other prehospital services.

## Conclusion

Live mission observation with post-mission debriefing is a feasible alternative to in-situ simulation programs in HEMS when missions must be prioritised over simulation training. This concept helps maximise the use of the facilitator as a resource to facilitate learning and reflection, irrespective of missions interrupting the in-situ simulation training. The facilitators do however need additional training beyond simulation facilitation to handle the live mission observation setting and their role in the live missions must be clear.

## Supplementary Information

**Additional file 1.**

**Additional file 2.**

## Data Availability

The dataset used during the study is available in an anonymous form from the corresponding author on a reasonable request. The recorded interviews have been deleted.

## Abbreviations

HEMS: Helicopter emergency medical services
SAR: Search and rescue
HTCM: HEMS technical crew member
PEARLS: Promoting excellence and reflective learning in simulation
VAS: Visuel analogue scale
SD: Standard deviation
