# Supplementary material for: Combining in-situ simulation and live HEMS mission facilitator observation: a flexible learning concept
Source: BMC Med Educ. 2021 Nov 15;21:579. doi: 10.1186/s12909-021-03015-w (PMC8594198; doi:10.1186/s12909-021-03015-w)
Supplement: Supplementary file 1 — Additional file 1. [file 12909_2021_3015_MOESM1_ESM.doc]

**Simulation**

1: I strongly agree Profession:

2: Agree Pilot ____

3: Partly agree HCM ____

4: Neutral Doctor ____

5: Partly disagree Nurse ____ (Ålesund)

6: Disagree

7: I strongly disagree

**Format of the training**

| Sufficient time was allotted for joint simulation training | | | | | | |
| --- | --- | --- | --- | --- | --- | --- |
| 1 | 2 | 3 | 4 | 5 | 6 | 7 |
| The simulation training was completed without interrupting other on-call duties | | | | | | |
| 1 | 2 | 3 | 4 | 5 | 6 | 7 |
| There was enough equipment available to make the simulation training realistic | | | | | | |
| 1 | 2 | 3 | 4 | 5 | 6 | 7 |
| I felt comfortable with the way the simulation training was set up | | | | | | |
| 1 | 2 | 3 | 4 | 5 | 6 | 7 |
| I felt comfortable with exposing my skills and competencies during the simulation training | | | | | | |
| 1 | 2 | 3 | 4 | 5 | 6 | 7 |

| Simulation was a realistic way to train |
| --- |

| 1 | 2 | 3 | 4 | 5 | 6 | 7 |
| --- | --- | --- | --- | --- | --- | --- |

**Content**

| The topic of the simulation training is relevant for this kind of simulation training | | | | | | |
| --- | --- | --- | --- | --- | --- | --- |
| 1 | 2 | 3 | 4 | 5 | 6 | 7 |
| This type of simulation training is useful for my occupational category | | | | | | |
| 1 | 2 | 3 | 4 | 5 | 6 | 7 |
| The simulation scenario was representative of the SOPs we trained on | | | | | | |
| 1 | 2 | 3 | 4 | 5 | 6 | 7 |
| The topic of the scenario training was relevant to the mission profile of the base | | | | | | |
| 1 | 2 | 3 | 4 | 5 | 6 | 7 |

Feedback

| The feedback after the simulation training was useful | | | | | | |
| --- | --- | --- | --- | --- | --- | --- |
| 1 | 2 | 3 | 4 | 5 | 6 | 7 |
| Enough time was allotted for feedback after the simulation training | | | | | | |
| 1 | 2 | 3 | 4 | 5 | 6 | 7 |
| It was easy to motivate oneself to complete the simulation training | | | | | | |
| 1 | 2 | 3 | 4 | 5 | 6 | 7 |

**Overall**

| I have a positive attitude towards this kind of training | | | | | | |
| --- | --- | --- | --- | --- | --- | --- |
| 1 | 2 | 3 | 4 | 5 | 6 | 7 |

Report how you experienced the training today from «0» completely unsatisfactory to «100» maximum satisfaction (mark with an “X”)

0 I--------------------------------------------------------------------------I 100

**Mission observation**

1: I strongly agree Profession:

2: Agree Pilot ____

3: Partly agree HCM ____

4: Neutral Doctor ____

5: Partly disagree Nurse ____ (Ålesund)

6: Disagree

7: I strongly disagree

**Format of the training**

| Sufficient time was allotted for debriefing and feedback after the mission observation | | | | | | |
| --- | --- | --- | --- | --- | --- | --- |
| 1 | 2 | 3 | 4 | 5 | 6 | 7 |
| The live mission observation was completed without interrupting other on-call duties | | | | | | |
| 1 | 2 | 3 | 4 | 5 | 6 | 7 |
| I am comfortable with being observed by a peer during a live mission | | | | | | |
| 1 | 2 | 3 | 4 | 5 | 6 | 7 |
| I felt comfortable with the way the live mission observation was carried out | | | | | | |
| 1 | 2 | 3 | 4 | 5 | 6 | 7 |
| I felt comfortable with exposing my skills and competencies during a live mission | | | | | | |
| 1 | 2 | 3 | 4 | 5 | 6 | 7 |

| Live mission observation with debriefing and feedback gives me the same benefits as simulation training |
| --- |

| 1 | 2 | 3 | 4 | 5 | 6 | 7 |
| --- | --- | --- | --- | --- | --- | --- |

**Content**

| The mission characteristics were well suited for mission observation with consequent debriefing and feedback | | | | | | |
| --- | --- | --- | --- | --- | --- | --- |
| 1 | 2 | 3 | 4 | 5 | 6 | 7 |
| Live mission observation with feedback and debriefing is useful for my occupational category | | | | | | |
| 1 | 2 | 3 | 4 | 5 | 6 | 7 |
| The facilitator managed to create a learning environment by relating elements from the mission observation to our SOPs | | | | | | |
| 1 | 2 | 3 | 4 | 5 | 6 | 7 |
| The execution of the mission was not disrupted by the peer joining for mission observation | | | | | | |
| 1 | 2 | 3 | 4 | 5 | 6 | 7 |

Feedback

| The debriefing and feedback after the mission observed was useful | | | | | | |
| --- | --- | --- | --- | --- | --- | --- |
| 1 | 2 | 3 | 4 | 5 | 6 | 7 |
| Enough time was allotted for debriefing and feedback after the mission observation | | | | | | |
| 1 | 2 | 3 | 4 | 5 | 6 | 7 |
| It was easy to motivate oneself to complete the mission with mission observation | | | | | | |
| 1 | 2 | 3 | 4 | 5 | 6 | 7 |

**Overall**

| I have a positive attitude towards this kind of training | | | | | | |
| --- | --- | --- | --- | --- | --- | --- |
| 1 | 2 | 3 | 4 | 5 | 6 | 7 |

Report how you experienced the training today from «0» completely unsatisfactory to «100» maximum satisfaction (mark with an “X”)

0 I--------------------------------------------------------------------------I 100
