# Supplementary material for: Combining in-situ simulation and live HEMS mission facilitator observation: a flexible learning concept
Source: BMC Med Educ. 2021 Nov 15;21:579. doi: 10.1186/s12909-021-03015-w (PMC8594198; doi:10.1186/s12909-021-03015-w)
Supplement: Supplementary file 2 — Additional file 2. [file 12909_2021_3015_MOESM2_ESM.docx]

# Interview guide:

# Facilitators being interviewed

Intro/preparation

You have the right to withdraw at any time. There is full anonymity (receives respondent number). You have the right to have the interview deleted (cannot change parts of the interview).

Interview

# Details:

What is your formal and informal experience/knowledge/training around medical simulation?

How long have you been working with medial simulation training?

Where did you do medical simulation training?

Where do you work now?

How old are you?

How many years do you have the job as an air ambulance doctor?

How many years have you facilitated medical simulation?

What are we going to talk about?

In this conversation, we will hear about your thoughts around the differences in medical simulation and structured debriefing after observed missions in the HEMS service.

We need to talk about what you think is good and bad about both ways of training/learning/developing skills.

By medical simulation, we mean the introduction of whole crew simulation in HEMS services in Norway for the on-call crew.
With live mission observation, we mean the use of the facilitator observing and debriefing real missions.

We have previously identified the following three main groups of challenges :

1. Motivation among participants
2. Hyppighed of simulations/training sessions
3. Getting it delivered

-----

Can you describe the differences in the experience of simulation with debriefing and live mission observation with a debriefing?

Are there differences in learning outcomes? (for individuals and crews)

Can you give examples of the before mentioned differences?

If you were to choose only one method of learning – which of these two would you choose?

How did it feel to be observing colleagues work?

Do you find it as different/challenging to do debriefings after observing live missions?

How did it not feel to interfere in the mission?

1. Motivation

Is there a difference in the motivation of the on-call crew between simulation and debriefing after live mission debriefings?

# Frequency

How often is it appropriate to offer simulation or live mission observation and debriefing?

Should there be an equal distribution between these two forms of training?

1. Delivery

What are the challenges for delivering this form of training?

# Can you give examples of the before mentioned?

# Interview guide

# Crewmembers being interviewed

Intro/preparation

You have the right to withdraw at any time. There is full anonymity (receives respondent number). You have the right to have the interview deleted (cannot change parts of the interview).

Interview

# Details:

What is your formal and informal experience/knowledge/training around medical simulation?

How long have you been working with medial simulation training?

Where did you do medical simulation training?

Where do you work now?

How old are you?

How many years do you have the job as an air ambulance doctor?

How many years have you facilitated medical simulation?

What are we going to talk about?

In this conversation, we will hear about your thoughts around the differences in medical simulation and structured debriefing after observed missions in the HEMS service.

We need to talk about what you think is good and bad about both ways of training/learning/developing skills.

By medical simulation, we mean the introduction of whole crew simulation in HEMS services in Norway for the on-call crew.
With live mission observation, we mean the use of the facilitator observing and debriefing real missions.

We have previously identified the following three main groups of challenges :

1. Motivation among participants
2. Hyppighed of simulations/training sessions
3. Getting it delivered

-----

Can you describe the differences in the experience of simulation with debriefing and live mission observation with a debriefing?

Are there differences in learning outcomes? (for individuals and for crews)

Can you give examples of the before mentioned differences?

If you were to choose only one method of learning – which of these two would you choose?

How did it feel to be observed in your practice?

1. Motivation

Is there a difference in the motivation of the on-call crew between simulation and debriefing after live mission debriefings? Do you think that there are motivational differences between pilots, doctors and HEMS crew members? - can you elaborate on this?

# Frequency

How often is it appropriate to offer simulation or live mission observation and debriefing?

Should there be an equal distribution between these two forms of training?

1. Delivery

What are the challenges for delivering and implementing this form of training?

# Can you give examples of the before mentioned?

# Can there be examples of the above?

Can you describe how felt it was when a "base colleague" observed/facilitated the training/simulation?

Would it be a different experience and learning outcome if it was a leader (clinical lead of the service) who conducted the live mission observations or facilitated the simulation training?

Can you elaborate on the above?
Can you describe the outcome of the live mission debriefings?

How is the "utilization rate" of the facilitator in simulation training and observed mission and debriefing, respectively?

Do you wish this project could continue? – and can you justify your answer?

Thank you for your participation!
